# A comprehensive review of the SLMTA literature part 2: Measuring success

**DOI:** 10.4102/ajlm.v3i2.276

**Published:** 2014-11-03

**Authors:** Elizabeth T. Luman, Katy Yao, John N. Nkengasong

**Affiliations:** 1 International Laboratory Branch, Division of Global HIV/AIDS, Center for Global Health, US Centers for Disease Control and Prevention, Atlanta, Georgia, United States

## Abstract

**Background:**

Since its introduction in 2009, the Strengthening Laboratory Management Toward Accreditation (SLMTA) programme has been implemented in 617 laboratories in 47 countries.

**Objective:**

We completed a systematic review of the published literature on SLMTA. The review consists of two companion papers; this article examines quantitative evidence presented in the publications along with a meta-analysis of selected results.

**Methods:**

We identified 28 published articles with data from SLMTA implementation. The SLMTA programme was evaluated through audits based on a standard checklist, which is divided into 12 sections corresponding to the 12 Quality System Essentials (QSEs). Several basic service delivery indicators reported by programmes were also examined. Results for various components of the programme were reviewed and summarised; a meta-analysis of QSE results grouped by the three stages of the quality cycle was conducted for 126 laboratories in 12 countries.

**Results:**

Global programme data show improved quality in SLMTA laboratories in every country, with average improvements on audit scores of 25 percentage points. Meta-analysis identified Improvement Management as the weakest stage, with internal audit (8%) and occurrence management (16%) showing the lowest scores. Studies documented 19% – 95% reductions in turn-around times, 69% – 93% reductions in specimen rejection rates, 76% – 81% increases in clinician satisfaction rates, 67% – 85% improvements in external quality assessment results, 50% – 66% decreases in nonconformities and 67% increases in staff punctuality.

**Conclusions:**

The wide array of results reported provides a comprehensive picture of the SLMTA programme overall, suggesting a substantive impact on provision of quality laboratory services and patient care. These comprehensive results establish a solid data-driven foundation for program improvement and further expansion.

## Introduction

Quality laboratory services are critical for ensuring optimal patient care and comprehensive public health response; however, laboratories in resource-poor countries have been one of the most neglected components of health systems.^1^ The Strengthening Laboratory Management Toward Accreditation (SLMTA) programme was developed in an effort to improve the quality of laboratories throughout the developing world. It is a competency-based training programme designed to enable laboratories to implement practical quality management systems (QMS) and encourage continuous quality improvement.

Since its introduction in 2009, the SLMTA programme has been implemented widely throughout Africa, as well as in the Caribbean, Central and South America, and Southeast Asia.^2^ The primary focus of the programme thus far has been implementation and expansion; until recently, little attention has been paid to the systematic examination of programme results in order to guide programme improvement and decision making.

This systematic literature review aims to compile existing results from evaluations of the SLMTA programme into a comprehensive report, in order to provide a broad view of the programme and to identify directions for the future. Because of the large volume of information collected, the review has been published in two parts. In Part 1, published separately, we present content analysis of qualitative findings and identified strategic directions for future priorities.^3^ In this companion paper, we compile the quantitative data presented in the publications, examine scores and indicators, and conduct a meta-analysis of selected results in order to establish a solid, data-driven foundation for programme improvement and to help guide future implementation.

## Research methods and design

A comprehensive search of electronic bibliographic databases was performed, as described in Part 1.^3^ We included all published and in-press studies that discussed the SLMTA programme.

The standard SLMTA implementation model includes three workshops, each of which is followed by a period of several months for laboratories to implement improvement projects, usually with onsite support and mentorship.^2^ Laboratories implementing the SLMTA programme are evaluated through audits based on the Stepwise Laboratory Quality Improvement Process Towards Accreditation (SLIPTA) checklist.^4^ Audit scores are categorised into star ratings, with zero stars corresponding to a score of 0% – 54%, one star 55% – 64%, two stars 65% – 74%, three stars 75% – 84%, four stars 85% – 94%, and five stars 95% – 100%. The checklist items are divided into 12 sections that represent the 12 Quality System Essentials (QSEs) as defined by the Clinical and Laboratory Standards Institute (CLSI).^5^ These QSEs can be grouped by stages of the quality cycle: Resource Management (equipment; facilities and safety; organisation and personnel; purchasing and inventory), Process Management (client management; documents and records; information management; process control and internal/external quality assessment) and Improvement Management (corrective action; internal audit; management reviews; occurrence management).^6^ To assess progress, baseline and exit audits are conducted before and after SLMTA implementation, respectively, using the SLIPTA checklist. ‘Surveillance’ audits are also often conducted after the exit audit in order to monitor continued improvement and assess sustainability.

Several studies provided scores by individual QSEs. We combined these data and conducted a meta-analysis in Microsoft^®^ Excel 2013 so as to determine common areas of strength, weakness and improvement. For studies reporting only median or mean QSE data for multiple laboratories, laboratory-level data were solicited from authors to further enhance the analysis. All cost estimates reported in local currency in published articles were converted into US dollars, based on the official exchange rate as of August 1, 2014. Percent changes in indicator results were calculated from published results if not reported directly in the papers.

## Results and discussion

### Literature search results

We identified 28 published articles on the SLMTA programme^2,7–33^ (Table 1). In total, these studies included detailed information on SLMTA implementation in 211 laboratories in 18 countries, as well as global summary data from all 617 laboratories in the 47 countries that have implemented SLMTA as of the end of 2013.

**TABLE 1:** Characteristics of published SLMTA studies.

Study	Country/Countries	Level of study	Number of laboratories	Years of study
Andiric et al.^7^	Tanzania	Select laboratory	1	2010–2011
Audu et al.^8^	Nigeria	Select laboratories	2	2010–2013
Eno et al.^9^	Cameroon	Select hospital	1	2011–2012
Gachuki et al.^10^	Kenya	Select laboratory	1	2010–2013
Guevara et al.^11^	Bahamas, Jamaica, Barbados, Trinidad and Tobago	One cohort	5	2011–2013
Hiwotu et al.^12^	Ethiopia	Two cohorts	45	2010–2012
Lulie et al.^13^	Ethiopia	Select laboratories	17	2013
Maina et al.^14^	Kenya	Select laboratories	5	2011–2012
Makokha et al.^15^	Kenya	Select laboratories	8	2010–2011
Maruta et al.^16^	NA	Global	NA	2009–2013
Maruti et al.^17^	Kenya	Select laboratory	1	2011–2013
Masamha et al.^18^	Mozambique	One cohort	8	2010–2012
Mataranyika et al.^19^	Namibia	One cohort	6	2012–2013
Mokobela et al.^20^	Bostwana	One cohort	7	2010–2011
Mothabeng et al.^21^	Lesotho	Two cohorts	18	2010–2011
Ndasi et al.^22^	Cameroon	One cohort	5	2009–2012
Nguyen et al.^23^	Vietnam and Cambodia	General	NA	2012–2013
Nkengasong et al.^24^	NA	General	NA	NA
Nkrumah et al.^25^	Ghana	Three cohorts	15	2011–2013
Nkwawir et al.^26^	Cameroon	Select laboratory	1	2009–2013
Noble et al.^27^	NA	General	NA	NA
Ntshambiwa et al.^28^	Bostwana	Select laboratory	1	2010–2013
Nzabahimana et al.^29^	Rwanda	Three cohorts	15	2010–2013
Nzombe et al.^30^	Zimbabwe	One cohort	19	2010–2012
Shumba et al.^31^	Zimbabwe	Two cohorts	30	2010–2012
Yao et al.^32^	NA	General	NA	NA
Yao et al.^2^	NA	General	NA	2009–2013
Yao et al.^33^	47 countries*	Global	617	2010–2013

*Source*: Luman, Yao and Nkengasong

SLMTA, Strengthening Laboratory Management Toward Accreditation; NA, not applicable.

* Angola, Antigua, Bahamas, Barbados, Belize, Botswana, Burundi, Cambodia, Cameroon, Columbia, Costa Rica, Cote d’Ivoire, Democratic Republic of the Congo, Dominica, Dominican Republic, El Salvador, Ethiopia, Ghana, Grenada, Guatemala, Haiti, Honduras, Jamaica, Kenya, Lesotho, Malawi, Mozambique, Namibia, Nicaragua, Nigeria, Panama, Peru, Rwanda, Sierra Leone, South Africa, South Sudan, Saint Kitts, Saint Lucia, Saint Vincent, Suriname, Swaziland, Tanzania, Trinidad and Tobago, Uganda, Vietnam, Zambia, Zimbabwe.

### Global programme results

Data from all laboratories implementing the SLMTA programme were collated and summarised in a single paper describing the global results of the programme to date.^33^ In total, 617 laboratories in 47 countries on four continents have implemented SLMTA in 65 training cohorts, with nearly 2000 laboratory staff trained in the programme. Most of the laboratories were at the district (38%), regional (27%) or national (18%) levels. The authors report that the starting level of laboratory quality in developing countries was very low, with 84% of SLMTA laboratories scoring below the one-star level at baseline. The 302 laboratories that had completed the programme had an average improvement of 25 percentage points; 70% achieved at least one star at exit audit and 22% of laboratories increased three or more star levels.

Estimates of the number of laboratory tests conducted by SLMTA laboratories suggested that the 617 laboratories enrolled in SLMTA conduct more than 100 million tests annually and that whilst only 16% of these tests were conducted by laboratories with at least one quality star *before* SLMTA, 68% were done by laboratories with at least one star *after* SLMTA implementation. That translates to approximately 58 million tests conducted by laboratories with little to no QMS prior to SLMTA which now have at least a basic quality system in place.^33^

### Quality System Essentials meta-analysis

Examining individual SLIPTA checklist scores for each of the 12 QSEs enables laboratories to pinpoint strengths, weaknesses and areas of improvement. QSE data have not been compiled systematically on a global scale. From the published papers, QSE data were presented for 126 laboratories in 12 countries.^8,11,12,14,15,18,20,21,22,25,26^ Individual studies reported substantial variability in high- and low-scoring QSEs. For example, some laboratories scored 0% for five of the 12 QSEs at exit audit, whereas others scored 100% for the same five QSEs.

**FIGURE 1:**
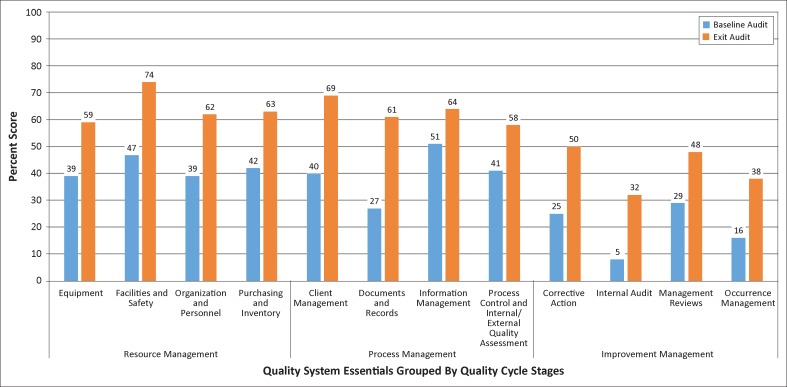
Baseline and exit audit scores for Quality System Essentials grouped by quality cycle stage from 126 laboratories in 12 countries.

At baseline, the weakest areas overall were in the Improvement Management stage of the quality cycle, including internal audit (5%), occurrence management (16%), corrective action (25%) and management reviews (29%) (Figure 1). At an average of 20%, this stage scored less than half of the other two stages, namely, Resource Management (42%) and Process Management (40%). None of the 12 QSEs had mean baseline scores above 55%; the highest scores were in information management (51%), facilities and safety (47%), purchasing and inventory (42%) and process control and internal/external quality assessment (41%).

At the exit audit, the four Improvement Management QSEs still showed the lowest scores, ranging from 32% – 50% (average 42%) (Figure 1). The Resource Management and Process Management stages had higher scores ranging from 58% – 74% (average 65% for Resource Management and 63% for Process Management). The greatest improvements were in documents and records (34 percentage points), client management (29 percentage points), and facilities and safety (27 percentage points). Each of the three stages had the same average improvement of 23 percentage points.

Based on results from five laboratories, Maina et al. found that the laboratories with the greatest overall score increases had focused on internal audit and corrective action; they then hypothesised that an improvement in these areas may be a catalyst for overall improvement in other areas.^14^ Meta-analysis results suggest that the corrective action QSE may be the most predictive of overall improvement; laboratories in the top quartile of overall improvement outperformed those in the bottom quartile by 62 percentage points for the corrective action QSE, compared to a median of 40 percentage points for the other QSEs. CLSI defines corrective action as an ‘action to eliminate the (root) cause of a detected nonconformity or other undesirable situation’.^34^ In the SLIPTA checklist, corrective action is assessed through four questions about how the laboratory deals with occurrence reports, nonconformities and discordant results.^4^ The International Organization for Standardization (ISO) confirms the importance of corrective action, saying that ‘the corrective and preventive actions system is the most critical element for an efficient quality system’.^35^ Additional work is needed to verify priority areas of improvement, as well as to delineate the set of essential improvement projects that will result in meaningful laboratory quality improvement.

### Official WHO AFRO SLIPTA audits and accreditation

A July 2009 survey of accrediting body registers identified 340 accredited laboratories in sub-Saharan Africa; only 28 (8%) of these laboratories were located outside of South Africa and nearly all were private, parastatal or donor-supported research facilities.^36^ By early 2013, little progress had been made, with 380 laboratories accredited in the region; only 35 (9%) laboratories outside of South Africa were accredited and three quarters of the 49 countries in the region had no accredited laboratories.^37^ However, the impact of SLMTA is beginning to show; as of September 2014, six laboratories enrolled in SLMTA in Kenya, the Bahamas, Vietnam and Zimbabwe have been accredited, at a median of 31.5 months after starting the SLMTA programme.^10,11,33^ Several laboratories have been recommended for accreditation or are in the process of application.^11,18,20,28^ Ninety-seven SLMTA laboratories have received official WHO AFRO SLIPTA audits conducted by representatives from the African Society for Laboratory Medicine,^33^ including 11 laboratories in published reports included in this review.^7,18,25,26,29^

### Service delivery indicators

In addition to audit scores, many of the studies reported improvements for indicators reflecting testing and customer and clinician satisfaction (Table 2). Three studies reported reductions in turnaround time for testing,^10,20,28^ with times decreasing by 19% – 95%. Patient and clinician satisfaction were commonly measured using surveys. Four studies showed relative improvements in patient satisfaction ranging from 30% to > 100%,^9,10,25,28^ although in one laboratory complaints from patients increased, possibly as a result of staff attrition.^17^ Two studies reporting on clinician satisfaction found improvements of approximately 80%.^17,28^

Indicators for laboratory management and overall functioning also showed improvements (Table 2). One laboratory reported a 65% decrease in corrective actions,^10^ five laboratories in the Caribbean Region reported decreases in nonconformities of 50% – 66%^11^ and two laboratories showed improvements in external quality assessment results of 67% – 85%.^10,17^ In a Kenyan laboratory, staff punctuality increased 67% and the need for equipment repairs decreased 63%.^17^ A Botswana laboratory successfully reduced losses resulting from expired reagents from $18 000 in 2010 to $40 in 2013;^28^ and three studies showed reductions in specimen rejection rates of 69% – 93%.^10,17,25^ When SLMTA was adapted and implemented at a hospital in Cameroon, patient wait times decreased 67% – 83%, infection rates and stillborn rates decreased (83% and 80%, respectively) and the number of patients and hospital revenue increased.^9^

**TABLE 2:** Health service indicators associated with SLMTA implementation as reported in published studies.

Study	Indicator	Method of measurement	Comparison periods	Result reported	Percent improvement (calculated)
Eno et al.^9^	Patient wait time in the emergency ward	Maximum patient wait times from arrival to departure from emergency room, estimated by scanning log books	Not specified (before and after SLMTA implementation)	Decreased from > 3 hours to < 30 min	83%
	Maximum overall patient wait time	Maximum patient wait times from arrival to laboratory results, estimated by scanning log books	Not specified (before and after SLMTA implementation)	Decreased from 3 days to < 1 day	67%
	Patient satisfaction	Proportion of patient suggestion box forms submitted with positive comments	Not specified (before and after SLMTA implementation)	Increased from 15% to 60%	400%
	Staff awareness of quality improvement programmes	Estimated by hospital director after inquiries	Not specified (before and after SLMTA implementation)	Increased from 10% to 75%	750%
	Hospital hygiene	Proportion of toilets that were functional in the facility	Not specified (before and after SLMTA implementation)	Increased from 10% to 75%	750%
	Infection rate	Estimated by the theatre nurse	Not specified (before and after SLMTA implementation)	Decreased from 3% to 0.5%	83%
	Stillborn rate	Estimated by the midwife of the maternity ward using birth records	Not specified (before and after SLMTA implementation)	Decreased from 5% to < 1%	80%
	Number of patients	Estimated by hospital director	Not specified (before and after SLMTA implementation)	Increased (amount not specified)	Unknown
	Hospital revenue	Provided by hospital director	Not specified (before and after SLMTA implementation)	Increased from $1638 to $2047	25%
Gachuki et al.^10^	Turnaround time for viral load testing	Review of data in the laboratory information management system	2010 versus 2013	Decreased from 20 days to 6 days	70%
	Turnaround time for ELISA testing	Review of data in the laboratory information management system	2010 versus 2013	Decreased from 191 days to 10 days	95%
	Turn-around time for CD4 testing	Review of data in the laboratory information management system	2010 versus 2013	Decreased from 24 hours to 12 hours	50%
	Service interruption days per month due to equipment downtime and stock outs	Review of data in the laboratory information management system	2010 versus 2013	Decreased from 15 days to 0 days	100%
	Patient satisfaction	Patient complaints summarised from patient feedback forms	2010 versus 2013	Decreased complaints from12 to 5	58%
	Specimen rejections	Review of data in the laboratory information system	2010 versus 2013	Decreased from 133 to 9	93%
	Corrective actions and occurrence management	Analysis of corrective action forms and quarterly reports	2010 versus 2013	Decreased from 74 to 26	65%
	External Quality Assessment results	Average correct responses on External Quality Assessment panel tests	2010 versus 2013	Increased from 60% to 100%	67%
Guevara et al.^11^	Number of nonconformities	Count of nonconformities in five laboratories	At baseline and surveillance audits	Decreased from 100 to 50; 77 to 32; 93 to 32; 61 to 24; and 58to 23	50%, 58%, 66%, 61%, 60%
	Number of standard operating procedures completed	Count of procedures completed in five laboratories	NA	205, 456, 292, 735, and 141standard operating procedures	NA
Lulie et al.^13^	Stock outs	Anecdotal report from laboratory managers	Not specified (before and after SLMTA implementation)	Decreased (amount not specified)	Unknown
	Interruption of service resulting from equipment problems	Anecdotal report from laboratories	Not specified (before and after SLMTA implementation)	Minimised (amount not specified)	Unknown
Maruta et al.^16^	Utilisation rate among graduates from the training-of-trainers programme	Survey of 195 participants asking whether they had delivered at least one SLMTA training or were still involved in SLMTA programme activities	NA	92%	NA
	Effectiveness of training-oftrainers programme	Survey of 195 participants asking whether the training was effective in preparing them to implement programme	NA	97%	NA
Maruti et al.^17^	External Quality Assessment results	Average correct responses on External Quality Assessment panel tests for 33 analytes, 3 times per year	2010 versus 2013	Increased from 47% to 87%	85%
	Staff punctuality	Average overall percent of person-days that staff arrived on time for their shift, based on employee time clock data	2011 versus 2013	Increased from 49% to 82%	67%
	Clinician satisfaction	Proportion of forms submitted with complaints	2011 versus 2013	Complaints decreased from 83%to 16%	81%
	Patient satisfaction	Proportion of forms submitted with complaints	2012 versus 2013	Complaints increased from 3%to 22%	-700%
	Sample rejection rate	Average rejection rate	2011 versus 2013	Decreased from 12% to 3%	75%
	Equipment repairs needed	Number of equipment repairs in the laboratory	2011 versus 2013	Decreased from 40 to 15	63%
	Ability to repair equipment internally	Proportion of equipment repairs carried out by internal engineers versus external	2011 versus 2013	Increased from 20% to 80%	400%
Mokobela et al.^20^	Turnaround time for laboratory testing	Anecdotal report from laboratories	Not specified (before and after SLMTA implementation)	Decreased (amount not given)	Unknown
Nkrumah et al.^25^	Specimen rejection rates	Percentage of total number of samples rejected, averaged over four laboratories	2011-2013	Decreased from 32% to 10%	69%
	Patient satisfaction	Proportion of patient suggestion box forms submitted with positive comments, averaged over four laboratories	2011-2013	Increased from 25% to 70%	300%
Ntshambiwaet al.^28^	Turnaround time for haematology	Analysis of results from the Integrated Patient Management System	April – September 2011 versusOctober 2011 – March 2012	Decreased from 72 minutes to 58 minutes	19%
	Turnaround time for chemistry	Analysis of results from the Integrated Patient Management System	April – September 2011 versusOctober 2011 – March 2012	Decreased from 154 minutes to 86 minutes	44%
	Turnaround time for CSF	Analysis of results from the Integrated Patient Management System	April – September 2011 versusOctober 2011 – March 2012	Decreased from 152 minutes to 106 minutes	30%
	Turnaround time for pregnancy tests	Analysis of results from the Integrated Patient Management System	April – September 2011 versusOctober 2011 – March 2012	Decreased from 97 minutes to 46 minutes	52%
	Patient satisfaction	Proportion of patients indicating ‘good’ or ‘very good’ on survey forms	2011 versus 2013	Increased from 56% to 73%	30%
	Clinician satisfaction	Proportion of clinicians indicating ‘good’ or ‘very good’ on survey forms	2011 versus 2013	Increased from 41% to 72%	76%
	Reagent wastage	Calculated laboratory losses resulting from expired reagents	Fiscal year 2011 versus 2013	Decreased from $18 000 to $40	> 99%
	Number of standard operating procedures completed	Count of procedures completed	NA	154 standard operating procedures	NA

SLMTA, Strengthening Laboratory Management Toward Accreditation; ELISA, enzyme-linked immunosorbent assay; NA, not applicable; CSF, cerebrospinal fluid.

### Cost

The reported costs per laboratory of implementing various components of SLMTA have varied widely (Table 3). Much of this variability is because of differences in what was included in the cost estimates, as well as location-specific factors, such the price of fuel, salary levels and distances to participating laboratories. The estimated cost of conducting the three-workshop SLMTA series has ranged from $1482 per laboratory in Zimbabwe using local facilitators in a central location^31^ to $21 480 in Cameroon using decentralised training.^22^ Mentorship cost per laboratory has ranged from $5689 in Zimbabwe^30^ to $24 000 in Ghana.^25^ The cost of implementing improvement projects has ranged from $10 000 in Ghana^25^ to $36 500 in a Kenyan laboratory seeking accreditation.^10^

**TABLE 3:** Cost estimates of various components of SLMTA implementation as reported in published studies.

Study	Portion of programme evaluated	Included costs	Excluded costs	Category	Component	Estimated cost per laboratory (US$)
Gachuki et al.^9^	Post-SLMTA to achieve ISO 15189 accreditation	Fees paid to the accrediting body, improvement projects	In-kind mentorship, SLMTA implementation, staff time	Single laboratory	Accreditation fees	7000
					Improvement projects	29 500
					Total	36 500
Ndasi et al.^21^	Workshops	Lodging, per diem, transportation, training materials, food, venue hire	Other components of SLMTA implementation (mentorship, supervision, improvement projects, audits), salaries	Centralised	SLMTA workshops per participant	4225
					SLMTA workshops per laboratory	21 122
				Decentralised	SLMTA workshops per participant	895
					SLMTA workshops per laboratory	21 480
Nkrumah et al.^24^	Mentorship, workshops and improvement projects	Programme implementer costs for mentors’ salaries, SLMTA workshops, and improvement projects	Not indicated	Per laboratory	Mentorship	24 000
					SLMTA workshops	6000
					Improvement project support	10 000
					Total	40 000
Nzombe et al.^28^	Mentorship	Mentor training, salaries, travel, lodging, internet access, equipment	All other components of SLMTA implementation (workshops, improvement projects, audits, staff time)	Model 1: Laboratory Manger Mentorship after SLMTA (per laboratory)	Mentorship	5486
					Supervision	928
					Total	6414
				Model 2: One Week per Month Mentorship after SLMTA (per laboratory)	Mentorship	4761
					Supervision	928
					Total	5689
				Model 3: Cyclical Embedded Mentorship after SLMTA (per laboratory)	Mentorship	9137
					Supervision	464
					Total	9601
				Model 4: Cyclical Embedded Mentorship with SLMTA (per laboratory)	Mentorship	9137
					Supervision	464
					Total	9601
Shumba, et al.^30^	Workshops, supervision and audits; training of local facilitators	Direct costs borne by programme implementer: training equipment, training (facilities and materials), trainers and supervisors (transport, accommodation, per-diem and fees) and participants (transport, accommodation and per-diem)	In-kind contributions and salaries of local facilitators and trainees	External facilitators (per laboratory)	Baseline audits	227
					SLMTA workshops	3634
					Supervision	400
					Exit audits	1540
					Total	5801
				Internal facilitators (per laboratory)	Baseline audits	7
					SLMTA workshops	1372
					Supervision	74
					Exit audits	29
					Total	1482
					Facilitator training	4444
				Theoretical, external facilitators (per laboratory)	Total	4837
				Theoretical, internal facilitators (per laboratory)	Total, first cohort (includes facilitator training)	8396
					Total, subsequent cohorts	1263

SLMTA, Strengthening Laboratory Management Toward Accreditation; ISO, International Organization for Standardization.

Three studies have compared the cost of various SLMTA implementation models. One study of 19 laboratories in Zimbabwe found that mentorship and supervision costs for four different models were similar ($5689-$9601 per laboratory), recommending that ‘countries should carefully consider which mentorship model or models would be best suited to their individual situation’.^30^ Another study in Zimbabwe found that implementing SLMTA using local (in-country) facilitators is more expensive than external facilitators for the first SLMTA cohort because of the costs associated with conducting an in-country training-of-trainers; however, over the course of national scale-up in 120 laboratories, use of local facilitators would save the country nearly 50% ($580 000 vs. $322 000).^31^ A Cameroonian study found that the cost per laboratory of centralised training was approximately the same as decentralised training ($21 122 vs. $21 480, respectively); centralised training required less trainer time, whilst decentralised training allowed more staff to participate.^22^

No published studies to date have reported a thorough examination of the cost of implementing the entire SLMTA programme, including each of the major components (training of mentors, trainers and auditors; conducting SLMTA workshops; mentorship, supervisory visits and implementation of improvement projects; and conducting audits). In addition, a more extensive cost-benefit analysis taking into consideration the value of laboratorians’ time (i.e., opportunity cost) to participate in the programme and implement changes in the laboratory along with tangible and intangible benefits of the programme is needed.^31^

### Limitations to the study

This review is subject to several limitations. Firstly, whilst 28 studies on SLMTA were identified and summarised, these reflect only 18 (38%) of the 47 countries and 211 (34%) of the 617 laboratories that have implemented the programme. Their results may not be representative of the programme as a whole, or a comprehensive account of all laboratories’ experiences. Secondly, whilst audit results were available for all laboratories because of the use of the SLIPTA checklist, the other indicators presented here were available in few of the published studies; in addition, methodologies varied between the studies, limiting the ability to combine and compare results directly.

Authors of the studies published thus far also point out several limitations. Firstly, the SLMTA programme as a whole is too young to allow an assessment of the long-term sustainability of results.^14,33^ Secondly, all of the published studies were observational; several studies examining the effect of mentorship or training methodologies note that laboratories were not assigned randomly, but were rather selected purposively based on convenience or other programmatic considerations. Thus there may have been other factors that could account for some of the differences.^8,15,20,30^ Similarly, none of the studies included control laboratories upon which to base a comparison.^22^ Thirdly, there is a lack of consistency in the qualifications of auditors; whilst the SLIPTA checklist is designed to help standardise the audit process, some variability between auditors may remain.^8,29^ Finally, several authors noted that their published studies are based on a small number of laboratories^14,15,20,30^ and some indicators were either not measured systematically^9^ or not measured at baseline.^9,28^

### Conclusion

In their summary of global-level findings, Yao et al. point out that ‘few [*other*] management and leadership development programmes have been implemented on a such a large scale with results-oriented outcome measures’.^33^ The wide array of results reported provides a comprehensive picture of the SLMTA programme overall, suggesting a substantive impact on provision of quality laboratory services and patient care. The full potential of the programme can be realised only if the lessons learned lead to informed action among laboratory workers, healthcare providers and policy makers toward the ultimate goal of providing quality patient care.
